# Lip Sonoanatomy and Relevance to Aesthetic Filler Injections: A Pictorial Review

**DOI:** 10.1111/jocd.70164

**Published:** 2025-04-09

**Authors:** Wei‐Ting Wu, Ke‐Vin Chang, Ondřej Naňka, Hsiang‐Chi Chang, Vincenzo Ricci, Kamal Mezian, Levent Özçakar

**Affiliations:** ^1^ Department of Physical Medicine and Rehabilitation National Taiwan University Hospital Bei‐Hu Branch Taipei Taiwan; ^2^ Department of Physical Medicine and Rehabilitation National Taiwan University College of Medicine Taipei Taiwan; ^3^ Center for Regional Anesthesia and Pain Medicine Wang‐Fang Hospital, Taipei Medical University Taipei Taiwan; ^4^ Institute of Anatomy, Charles University, First Faculty of Medicine Prague Czech Republic; ^5^ Department of Physical Medicine and Rehabilitation Taipei Hospital Ministry of Health and Welfare New Taipei City Taiwan; ^6^ Physical and Rehabilitation Medicine Unit Luigi Sacco University Hospital, ASST Fatebenefratelli‐Sacco Milan Italy; ^7^ Department of Rehabilitation Medicine Charles University, First Faculty of Medicine and General University Hospital in Prague Prague Czech Republic; ^8^ Department of Physical and Rehabilitation Medicine Hacettepe University Medical School Ankara Turkey

**Keywords:** aesthetic medicine, intervention, labial artery, mouth, ultrasonography

## Abstract

**Background:**

Lips are central to facial aesthetics, influencing overall balance and harmony. Ultrasound has become a key tool for assessing lip musculature and neurovascular structures, particularly in aesthetic filler injections. By enhancing precision and safety, ultrasound is valuable in procedures addressing age‐related lip changes, yet standardized scanning protocols remain underexplored.

**Aims:**

This review examined lip anatomy, summarized ultrasound applications in aesthetic procedures, introduced a structured scanning protocol, and highlighted its role in guiding filler injections.

**Methods:**

A systematic search of PubMed, Scopus, Embase, and Web of Science was conducted up to August 1, 2024, using keywords related to ultrasound and lip anatomy. Studies involving human subjects or cadavers using ultrasound for lip assessment and injection guidance were included, whereas nonhuman studies, alternative imaging methods, and research unrelated to the lip region were excluded. Six studies met the criteria.

**Results:**

Ultrasound improves precision in lip injections by identifying key structures, including the superior and inferior labial arteries and the orbicularis oris muscle, reducing vascular risks. A structured scanning approach enhances procedural safety and efficacy.

**Conclusions:**

Ultrasound is a valuable tool in aesthetic lip procedures, improving accuracy and minimizing complications. Further research is needed to refine protocols and establish ultrasound as a standard practice in lip augmentation.

## 1 Introduction

Lips play a crucial role in facial attractiveness, significantly contributing to overall aesthetic balance [1]. Full, well‐defined lips are often associated with youthfulness and sensuality, enhancing facial expressions, conveying emotions, and impacting social interactions [1]. Lips define the contours of the lower face, complementing other facial features such as the eyes and nose. Their symmetry, volume, and shape are key factors influencing perceptions of appeal. On the other hand, ultrasound (US) has become a valuable tool in evaluating the musculature [2] and neurovascular structures [3] of the face, including the lips, particularly in the context of aesthetic filler injections. Hyaluronic acid fillers are widely used for restoring lip volume and achieving defined, full lips—a common goal in cosmetic practice. However, such fillers, especially those with a high degree of cross‐linking, are more immunogenic and have been associated with delayed‐onset immune‐mediated nodules [4].

Age‐related changes, such as drooping, thinning of the lips, and lip lengthening, are often addressed through aesthetic procedures [5]. Understanding lip anatomy and mastering US scanning techniques are paramount for effective treatment. Using color Doppler, practitioners can identify the labial artery to avoid vascular complications. Proper filler placement is essential to prevent tissue compromise or vascular issues. In cases of overfilled lips, hyaluronidase injections can also be used for correction [6]. Despite the benefits, complications, such as vascular occlusion, infection, and granuloma formation, can occur following filler injections; therefore, precise technique and thorough anatomical knowledge are very important. This pictorial review aims to (i) revisit the lip anatomy, (ii) provide an overview of the literature on lip US, (iii) present a systematic approach to lip ultrasonography, and (iv) demonstrate US guidance during aesthetic filler injections.

## 2 Anatomy

Both the upper and lower lips contain a mucosal membrane, vermilion, and cutaneous surfaces (perioral skin) [7]. The upper lip extends from the nasolabial folds to the inferior margin of the nose, whereas the lower lip encompasses the region between the lateral commissures and the labiomental crease (the natural indentation between the lower lip and the chin). The upper lip harbors the philtrum, a central depression bordered by paramedian vertical philtral ridges, located directly below the nasal septum. The inferior margin of the philtrum forms the downward arch of the cupid's bow, and the central protuberance of the upper lip is known as the tubercle or procheilon. The philtrum provides extra skin that can be utilized for movements involving the extension of the upper lip. The average horizontal length of the vermilion portion is longer in the upper lip than in the lower lip (8.0 vs. 7.5 cm on average). The lower lip should be slightly more protruded than the upper lip, with approximately 2–4 mm of teeth exposed [8].

**FIGURE 1:**
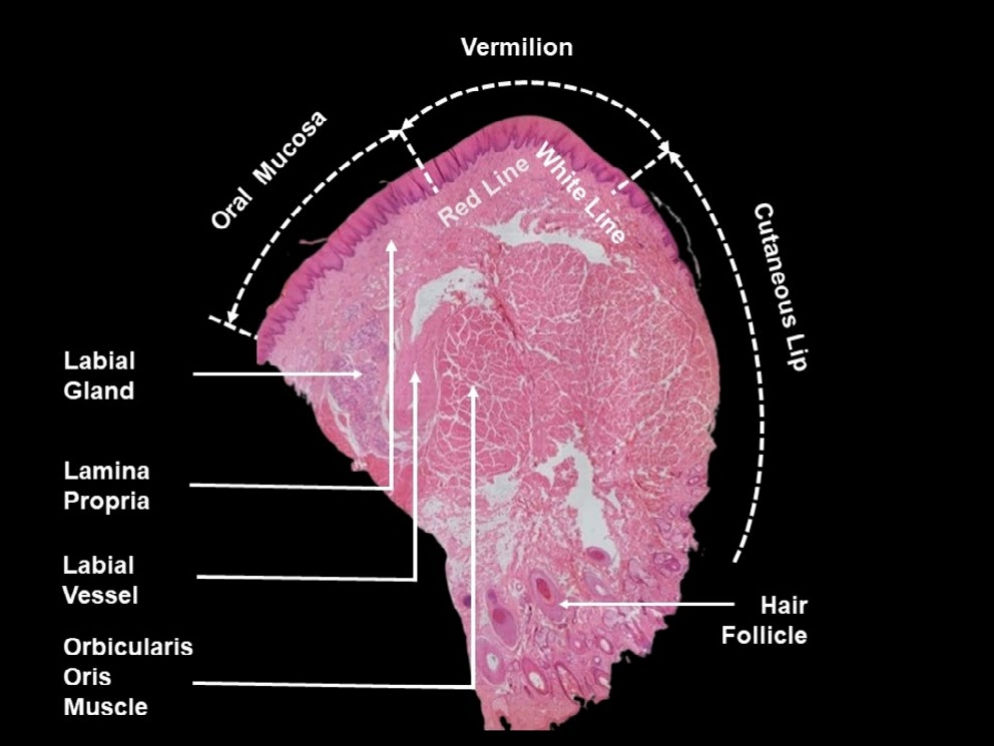
Histological (sagittal) section of the lip region.

The surface of the lip is known as the vermilion, a modified mucosa that transitions from the perioral skin (keratinized stratified squamous epithelium with sebaceous glands, sweat glands, and hair follicles) to the oral mucosa (nonkeratinized stratified squamous epithelium with a dense lamina propria and submucosa connective tissue). The lamina propria is a layer of connective tissue found just below the epithelial layer of the oral mucosa [9], housing numerous labial glands (minor salivary glands) within the inner mucosal lining of the lip. The vermilion consists of nonkeratinized stratified squamous epithelium and lacks typical skin appendages while being highly vascularized [10]. The limited number of melanocytes and the dense superficial vasculature beneath the membrane give the lips their reddish appearance. The red line marks the boundary between the vermilion and the mucous membrane of the oral cavity on both the upper and lower lips, whereas the white line (vermilion border) separates the facial skin from the vermilion (Figure 1).

**FIGURE 2:**
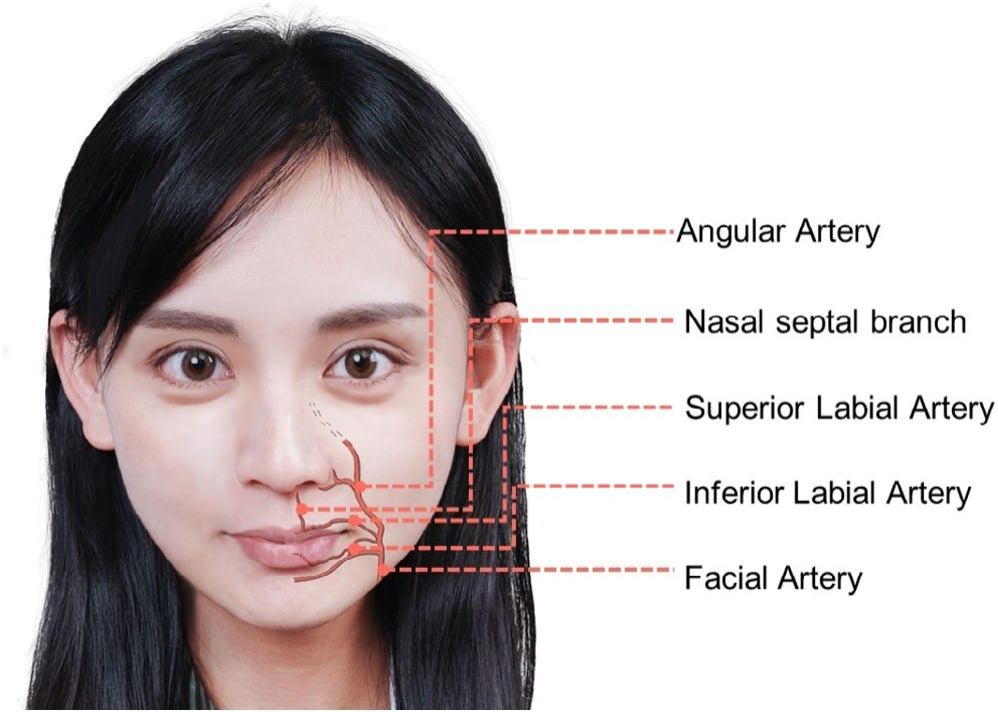
Illustration for the arteries supplying the lip region. The images represent an adaptation of our co‐author's (H.‐C.C) face with permission for publication.

The blood supply to the upper lip is provided by the superior labial artery, a branch that originates from the facial artery (Figure 2). In a cadaveric study by Lee et al. [11], the superior labial artery is classified into four types: Type I (56.7%), where both the artery and the alar branch, which also supplies blood to the nasal ala, arise independently from the facial artery; Type II (21.7%), where the superior labial artery branches from the facial artery and subsequently gives off an alar branch; Type III (15.0%), where it serves as the terminal branch of the facial artery; and Type IV (6.7%), where the artery is not found. Regarding the blood supply to the lower lip (Figure 2), findings from the cadaveric study of Edizer et al. [12] indicated that the inferior labial artery serves as the primary artery, consistently branching from the facial artery in all cases. Additionally, the authors noted the presence of the sublabial artery (in 71% of the cadavers) which may branch off from either the inferior labial or facial arteries. The study also identified end‐to‐end anastomosis between bilateral inferior labial arteries and anastomosis between the inferior labial artery and the submental artery in certain cases.

**FIGURE 3:**
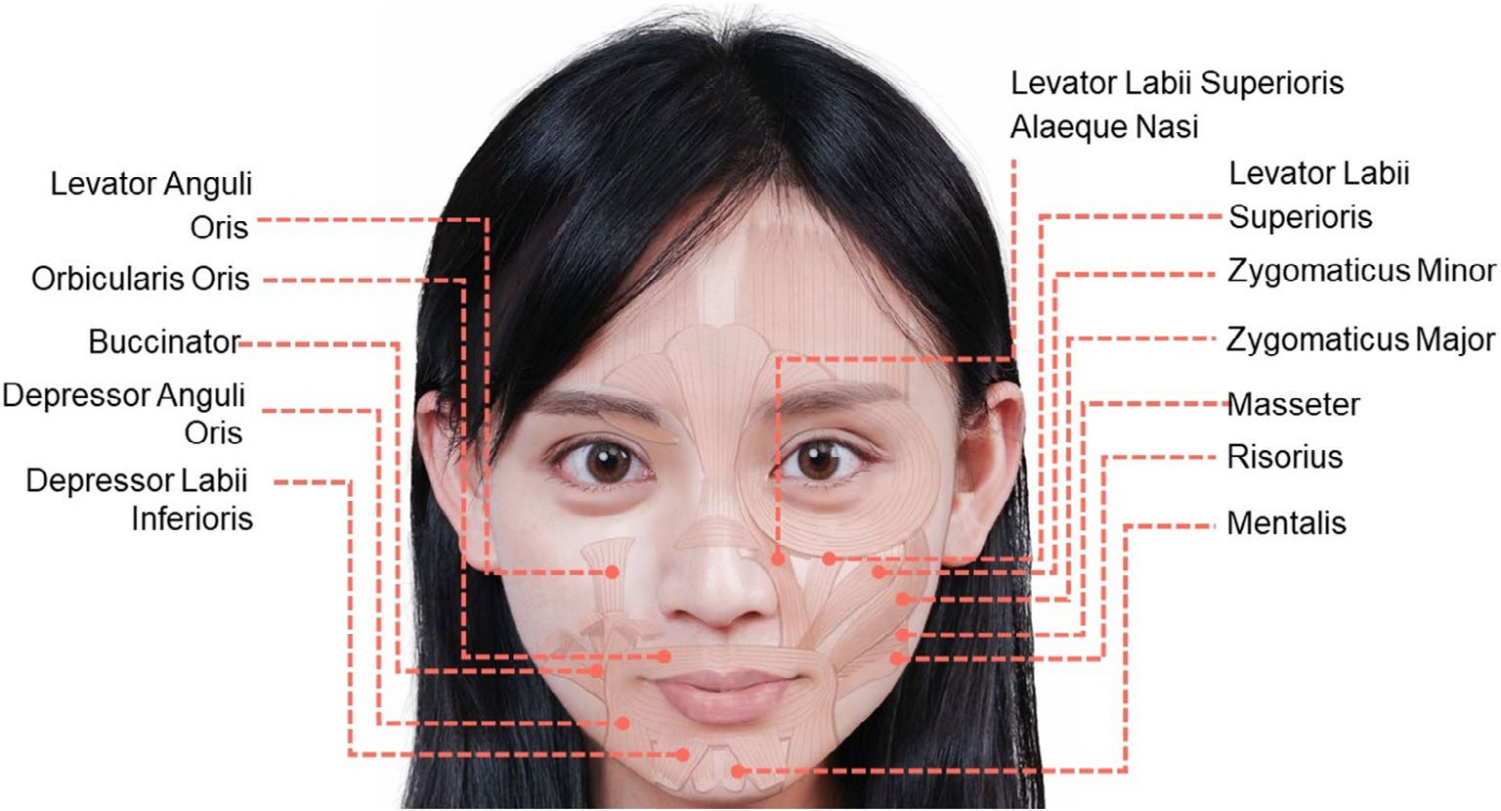
Illustration for the muscles surrounding the lip region. The images represent an adaptation of our co‐author's (H.‐C.C) face with permission for publication.

The modiolus, located at the corners of the mouth, serves as an attachment point for several muscles that contribute to facial expressions [2]. They include the orbicularis oris, which closes and puckers the lips; levator anguli oris, which lifts the angle of the mouth; zygomaticus major, which raises the lips for smiling; risorius, which extends the angle of the mouth laterally; buccinator, which aids in blowing and chewing by forming the wall of the cheek; and depressor anguli oris, which lowers the angle of the mouth for frowning (Figure 3). Additionally, there are lip‐relevant muscles not attaching to the modiolus: The zygomaticus minor, which elevates the upper lip and exposes the maxillary teeth; the levator labii superioris, which lifts the upper lip; levator labii superioris alaeque nasi, which raises the upper lip and the wing of the nose; depressor labii inferioris, which lowers the lower lip; and mentalis, which raises and protrudes the lower lip.

**FIGURE 4:**
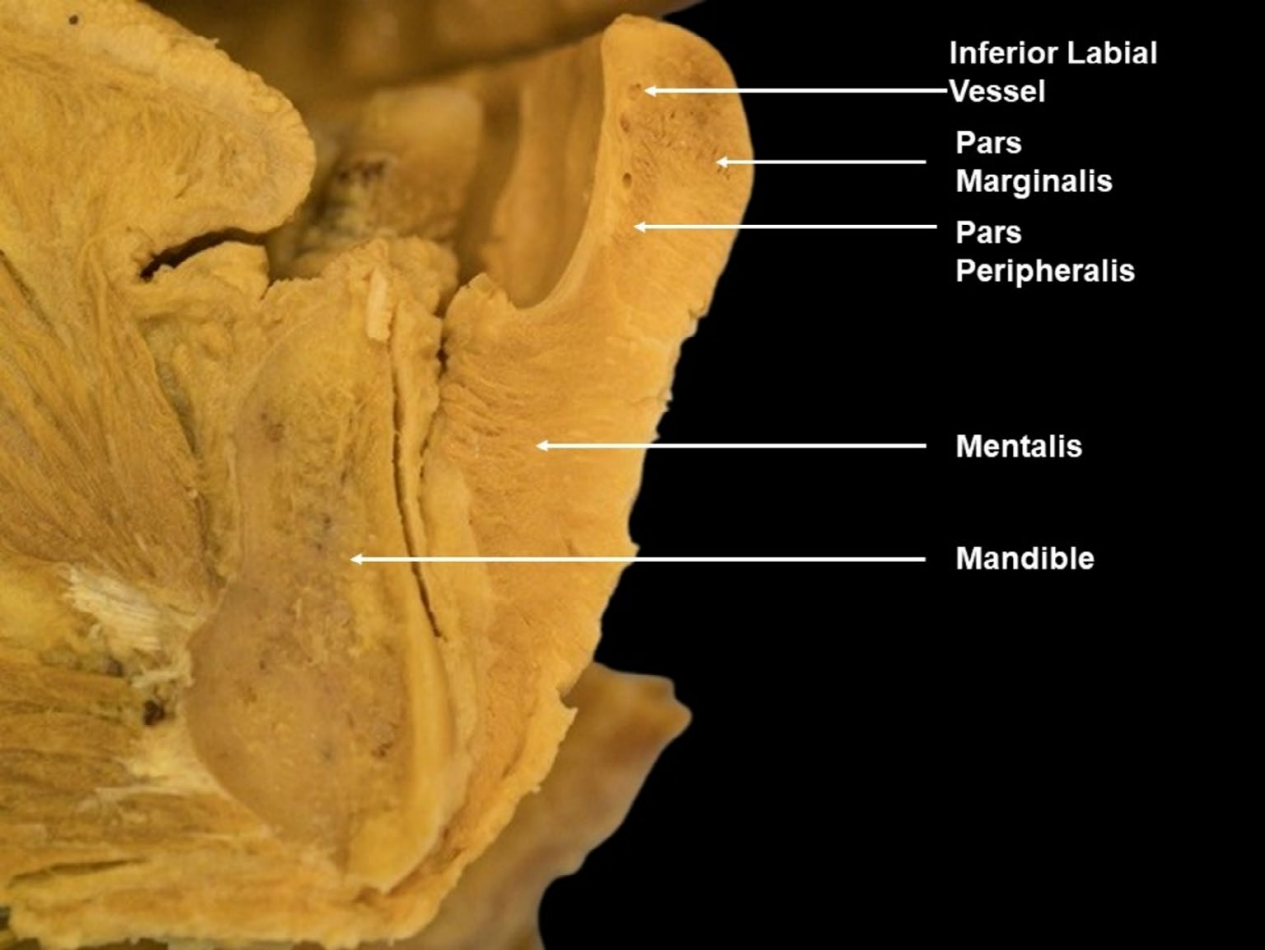
Cadaveric sagittal dissections showing the different components of the orbicularis oris muscle, including pars marginalis and peripheralis.

A noteworthy muscle is the orbicularis oris [13]. It consists of pars peripheralis, located on the inner aspect with concentric fibers at the perioral area, and pars marginalis, situated more externally just beneath the vermillion border of the upper and lower lips (Figure 4).

## 3 Literature Review

A structured approach was employed to identify pertinent research articles via four electronic databases: PubMed, Scopus, Embase, and Web of Science. The search period spanned from the inception of each database up to August 1, 2024—using the following combinations: (“ultrasound” or “sonography” or “ultrasonography”) and (“lip” or “labium” or “labia” or “face”). The inclusion criteria were: (1) research involving human subjects and cadavers, and (2) the use of US for the assessment and injection of the lip region. The exclusion criteria were: (1) research involving nonhuman subjects, (2) use of imaging methods other than US, (3) articles that did not address the lip region, and (4) studies primarily investigating the application of US for the evaluation of cleft lip/palate. A total of six articles met our criteria and were summarized as below:

In 1998, McAlister et al. [14] aimed to determine the differences in the thickness of the lip levator musculature between men and women as well as its relation to smile line height in adults. They studied 54 undergraduate dental students, grouped by smile line height, and measured muscle thickness using US. The study found that women had higher smile lines and thicker zygomaticus major muscles than men. However, there were no significant differences in muscle thickness among the different smile line height groups.

In 2005, Lydia et al. [15] used US to visualize the caliber‐persistent labial artery (CPLA) in three patients, comparing sonograms with clinical and histopathologic findings. The imaging showed significant dilatation of the labial artery, with its course being either vertical or oblique. They concluded that US and color Doppler imaging are effective for diagnosing, assessing, and monitoring labial lesions related to CPLA, potentially avoiding the need for diagnostic surgery in common pulsatile nodules.

In 2019, Mlosek et al. [16] reported a 43‐year‐old female patient who underwent an US scan 8 months after lip augmentation. The scan revealed various delayed side effects of the procedure, that is, increased echogenicity of the lip soft tissue, excessively deep filler injection, and blockage of the inferior labial artery (in contrast to the patent superior labial artery seen in the sagittal‐US view). This case report underscored the utility of US in assessing the unwanted side effects following lip filler administration.

In 2019, Tansatit et al. [17] conducted a study using translucent facial flaps to examine the origins of the inferior labial artery in 11 cadavers. They also used US to analyze this artery in 20 volunteers. The findings identified five distinct origins for the inferior labial artery: the labiomental, modiolar, ascending mental, facial, and superior labial arteries. The types of arterial origins from modiolar, facial, and superior labial arteries were noted as being particularly susceptible to injury during filler injections. US showed that the artery's average depth was 6 mm, and the vermilion border was pinpointed as a high‐risk area for injections.

In 2020, Cotofana et al. [18] conducted a study to improve the precision of labial soft tissue filler injections by mapping the spatial trajectories of the superior and inferior labial arteries. In 41 participants, they found that the most frequent location for both arteries was the submucosal plane (58.5%), followed by the intramuscular plane (36.2%)—that is inside the orbicularis oris muscle—and the subcutaneous plane (5.3%). The superior labial artery was typically found at an average depth of 5.6 mm in the upper lip, whereas the inferior labial artery was at an average depth of 5.2 mm in the lower lip. Both arteries were mainly situated within the vermilion zone.

In 2023, Lee et al. [19] used US to map the locations of superior and inferior labial arteries in relation to the vermilion border. In 60 volunteers without any facial treatment within the past 6 months, upper and lower lip thicknesses were measured as 9.4 ± 0.4 mm and 10.9 ± 0.7 mm, respectively. Labial arteries were primarily found in the wet mucosal layer of the upper (35%–57%) and lower (28%–55%) lips. The superior labial artery was also frequently located in the intramuscular layer of the upper lip (20%–45%), and the inferior labial artery was often observed at the dry–wet mucosal junction in the lower lip (5%–27%). The depth of the arteries was 5.3 ± 0.3 mm in the upper and 4.2 ± 0.4 mm in the lower lips. These findings suggest that lip augmentation procedures should use very superficial injections.

## 4 Sonography

**FIGURE 5:**
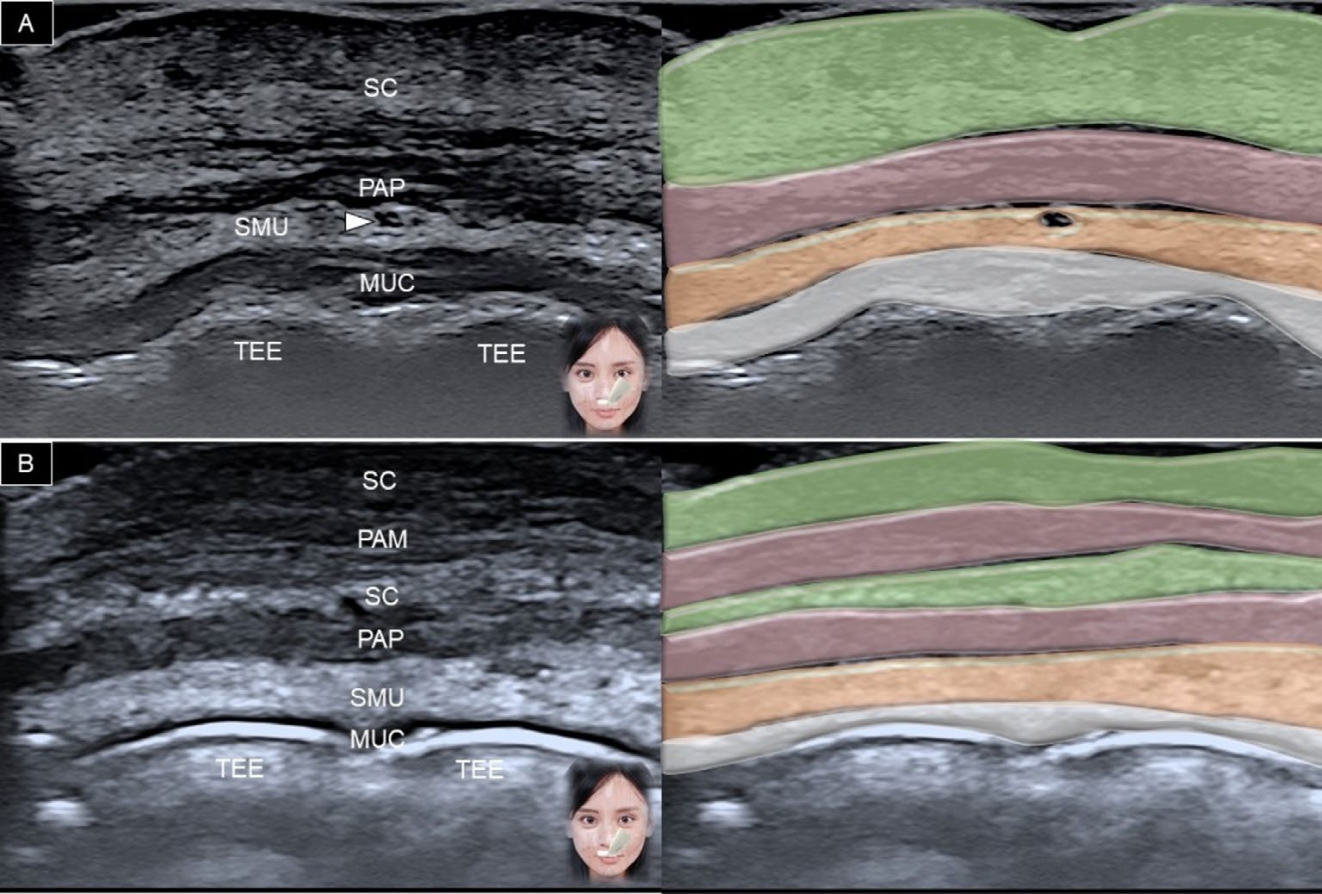
Ultrasound imaging and schematic illustration of the upper lip in the horizontal plane, capturing the area between the vermilion and dry skin (A) and over the vermilion (B). MUC, oral mucosa layer; PAM, pars marginalis; PAP, pars peripheralis; SC, subcutaneous layer; SMU, submucosal layer; TEE, teeth; white arrowhead, superior labial artery.

**FIGURE 6:**
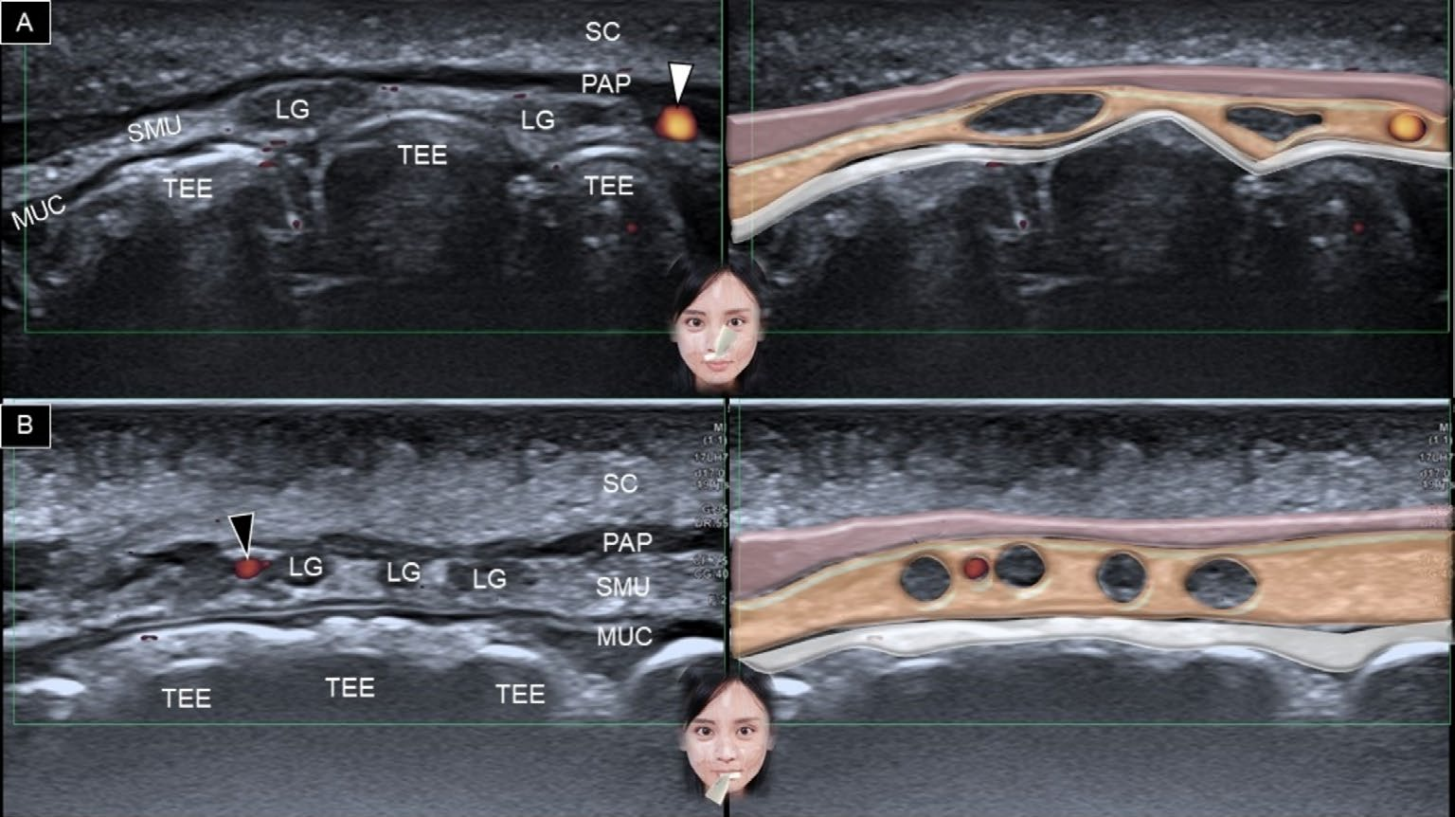
Ultrasound imaging and schematic illustration of the labial glands in the horizontal plane, showing the upper (A) and lower (B) lips. LG, labial gland; MUC, oral mucosa layer; PAP, pars peripheralis; SC, subcutaneous layer; SMU, submucosal layer; TEE, teeth, white arrowhead, superior labial artery; black arrowhead, inferior labial artery.

The transducer is placed horizontally over the philtrum and gradually moved to the vermilion border of the upper lip. A generous amount of gel is applied between the transducer's footprint and the skin to avoid any vascular compression. At the level of the white line (between the vermilion and dry skin), the following structures can be delineated from superficial to deep: dermis, subcutaneous fat, pars peripheralis, submucosal fat, and teeth (Figure 5). When the transducer is moved to the vermilion, the pars marginalis is seen intersecting the subcutaneous fat (Figure 6A). In the submucosal fat layer, hypoechoic round structures correspond to labial mucous glands [20]. Power Doppler mode can be used to visualize the superior labial artery, which is typically found in the submucosal layer.

**FIGURE 7:**
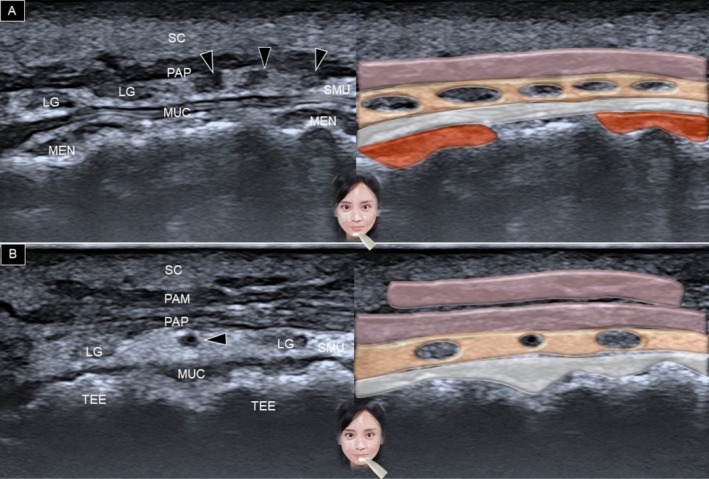
Ultrasound imaging and schematic illustration of the lower lip in the horizontal plane, depicting the region at the level of the labiomental crease (A) and over the vermilion (B). LG, labial gland; MEN, mentalis; MUC, oral mucosa layer; PAM, pars marginalis; PAP, pars peripheralis; SC, subcutaneous layer; SMU, submucosal layer; TEE, teeth, black arrowheads, inferior labial artery.

For the lower lip, the transducer is positioned in the horizontal plane along the labiomental crease (Figure 7A). The transducer is then moved cranially, and similar structures are recognized as in the upper lip region (Figure 7B). Instead of the superior labial artery, the arteries identified in the lower lip region are the inferior labial artery and its branches (Figure 6B) [3].

**FIGURE 8:**
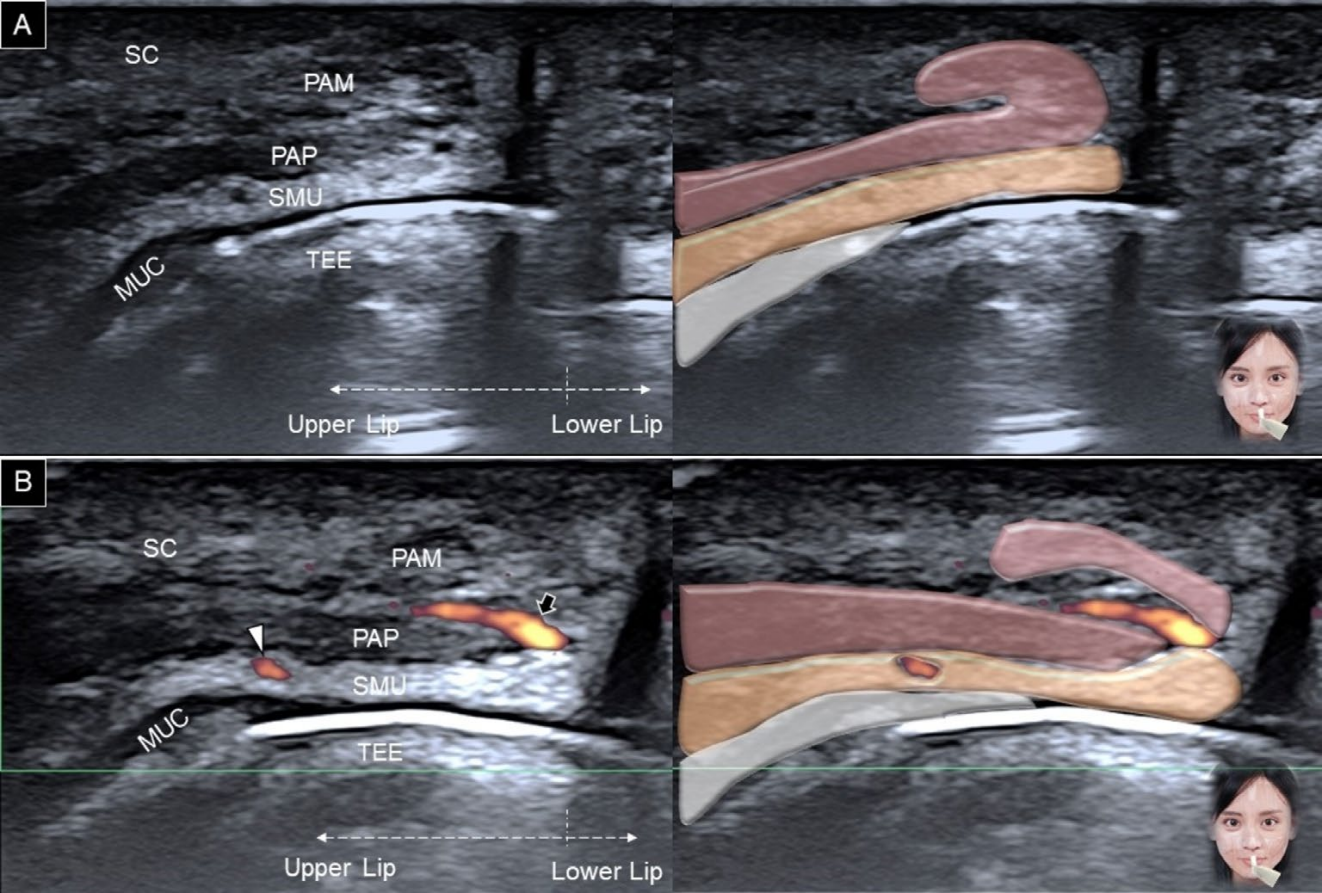
Ultrasound imaging and schematic illustration of the lip in the sagittal plane, focusing on the upper lip using B mode (A) and power Doppler (B). MUC, oral mucosa layer; PAM, pars marginalis; PAP, pars peripheralis; SC, subcutaneous layer; SMU, submucosal layer; TEE, teeth; white arrowhead, superior labial artery; black arrow, nasal septal branch.

The transducer is then redirected to the sagittal plane, crossing the upper and lower lips. The orbicularis oris appears as a J‐shaped structure (Figure 8A), with the pars marginalis forming the tip of the J and the pars peripheralis forming the stem [19]. When the transducer is positioned at the midline, the anastomosis of the main trunk of the superior labial artery and its nasal septal branch may be seen within the subcutaneous layer, situated between the pars marginalis and peripheralis (Figure 8B).

**FIGURE 9:**
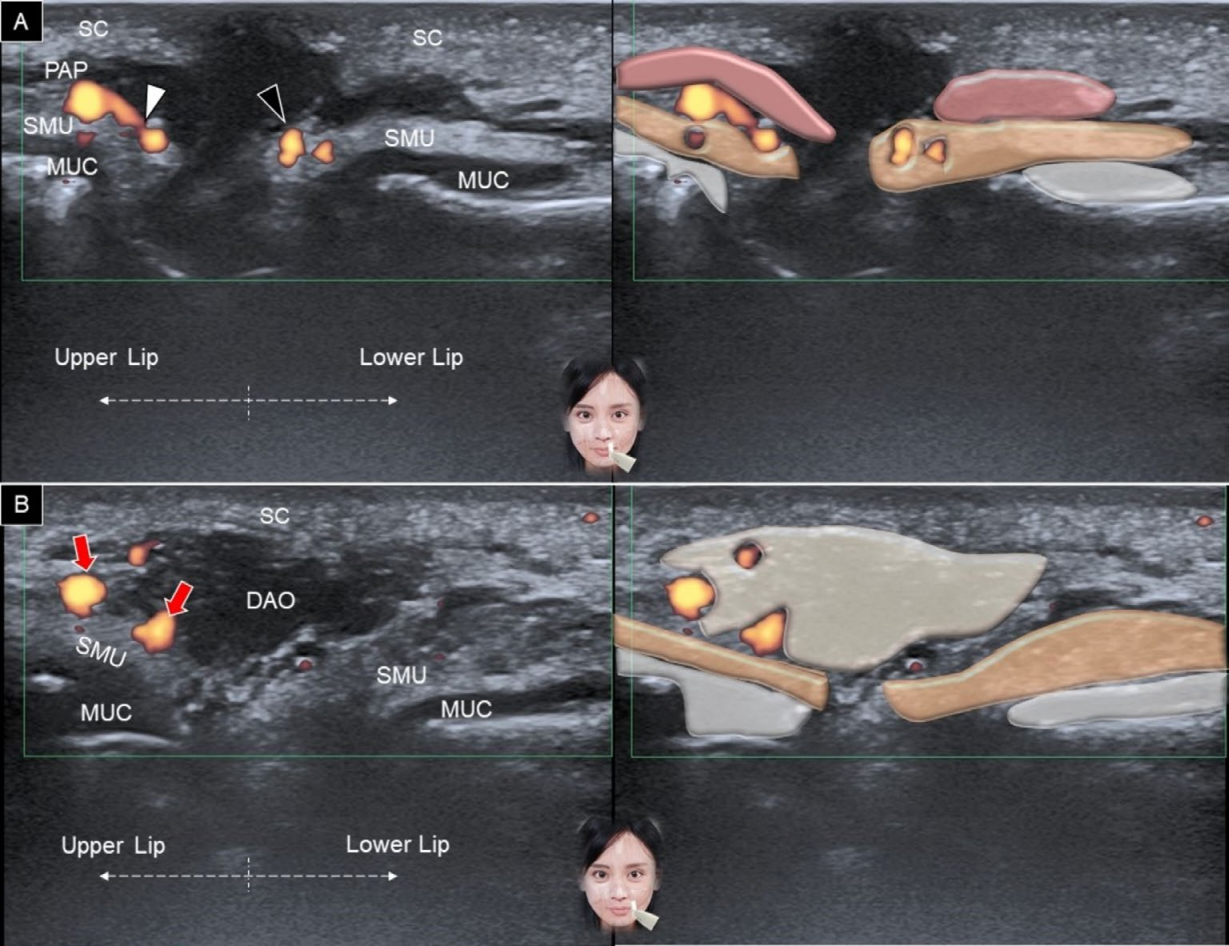
Power Doppler ultrasound imaging and schematic illustration of the lip in the sagittal plane, highlighting the superior and inferior labial arteries within the submucosal layer (A) and the facial artery located medial to the depressor anguli oris muscle (B). DAO, depressor anguli oris; MUC, oral mucosa layer; PAP, pars peripheralis; SC, subcutaneous layer; SMU, submucosal layer; white arrowhead, superior labial artery; black arrowhead, inferior labial artery; red arrows, facial artery.

The transducer is then moved from the midline to the oral commissure, initially revealing the superior or inferior labial arteries within the submucosal layer (Figure 9A). More laterally, the superior and inferior labial arteries typically branch from the facial artery within a layer medial to and deep to the depressor anguli oris muscle (Figure 9B). This finding aligns with the anatomical study by Tanvaa Tansatit et al. [21], which demonstrated that the superior and inferior labial arteries originate from the facial artery in a deep plane and become more superficial as they approach the midline.

**FIGURE 10:**
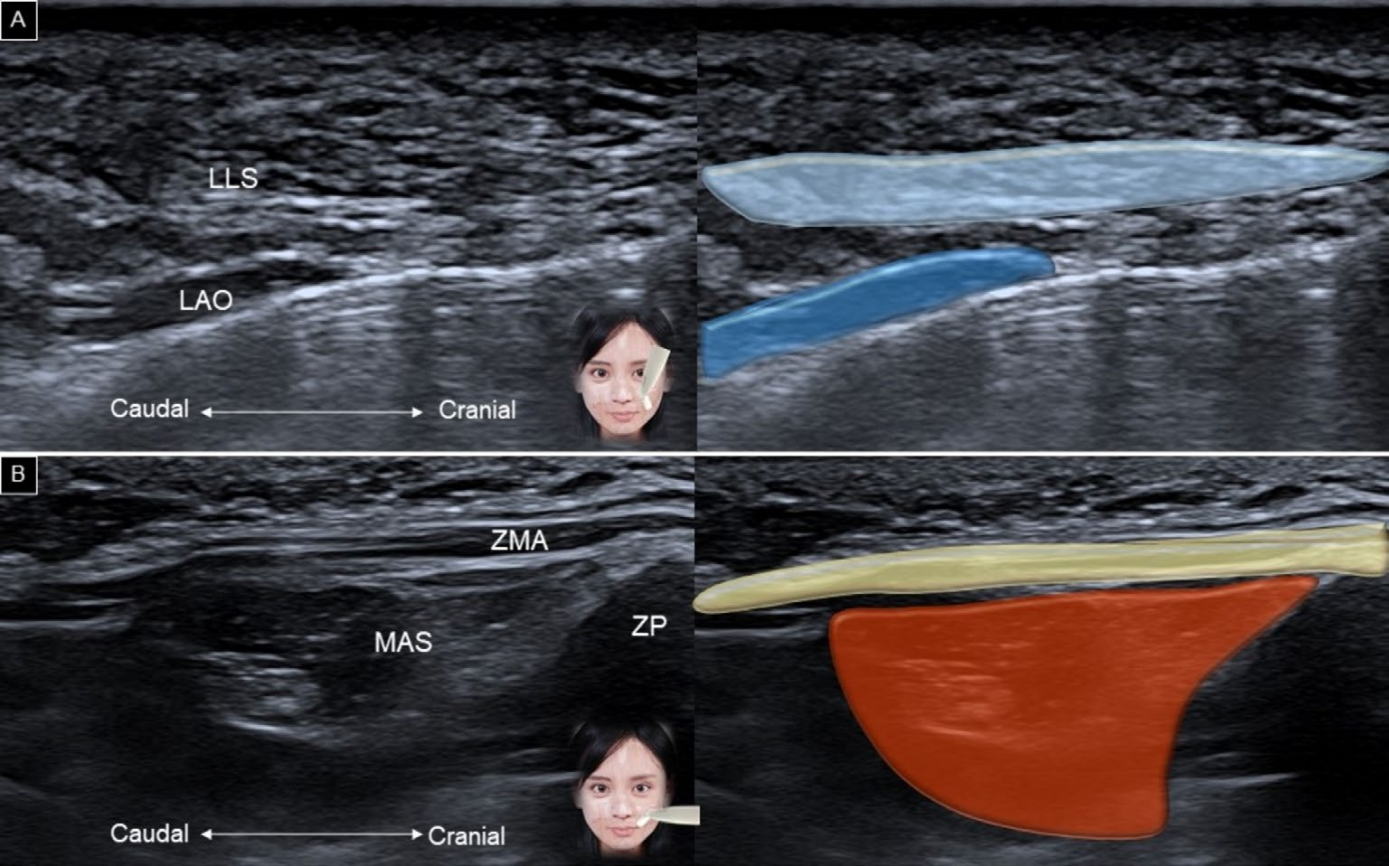
Ultrasound imaging and schematic illustration of the levator labii superioris and levator anguli oris (A), and zygomaticus major (B) muscles. LAO, levator anguli oris; LLS, levator labii superioris; MAS, masseter; ZMA, zygomaticus major; ZP, zygomatic process.

Muscles attaching to the modiolus can be scanned by fixing the medial part of the transducer over the angle of the mouth [2]. With the other end of the transducer pointed toward the infraorbital foramen, the long axis of the levator anguli oris can be visualized, with the levator labii superioris located above it (Figure 10A). Redirecting the other end of the transducer toward the zygomatic process of the maxilla reveals the long axis of the zygomaticus major (Figure 10B).

**FIGURE 11:**
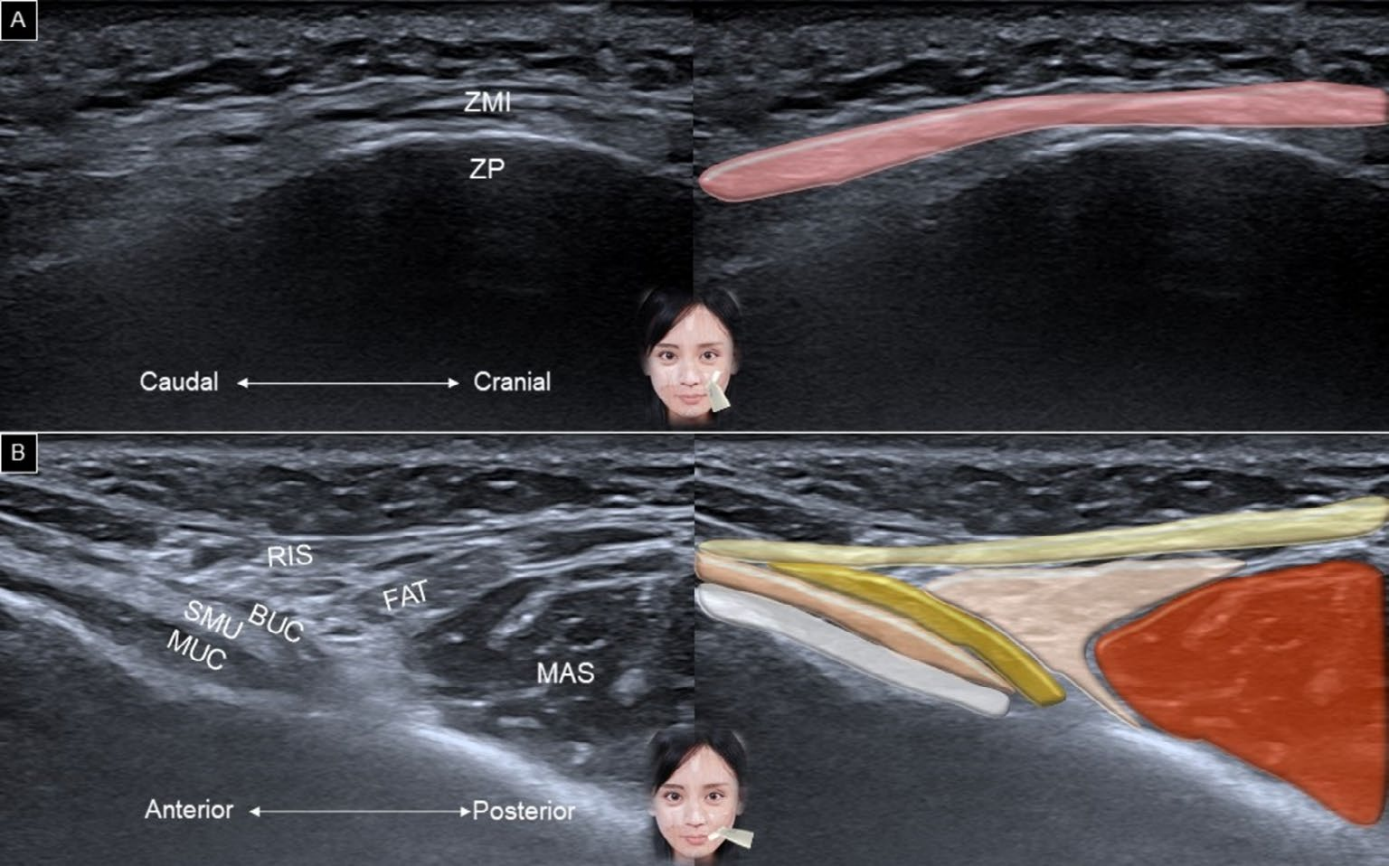
Ultrasound imaging and schematic illustration of the zygomaticus minor (A), risorius and buccinator (B) muscles. BUC, buccinator; FAT, buccal fat pad; MAS, masseter; MUC, oral mucosa layer; RIS, Risorius; SMU, submucosal layer; ZMI, zygomaticus minor; ZP, zygomatic process.

The zygomaticus minor follows a similar course to the zygomaticus major, but has a smaller muscle belly and originates more medially on the cranium (Figure 11A). When the transducer is adjusted along the horizontal plane, the risorius can be identified superficially, and the buccinator can be seen deeply, with the buccal fat pad interposed between the two muscles (Figure 11B).

**FIGURE 12:**
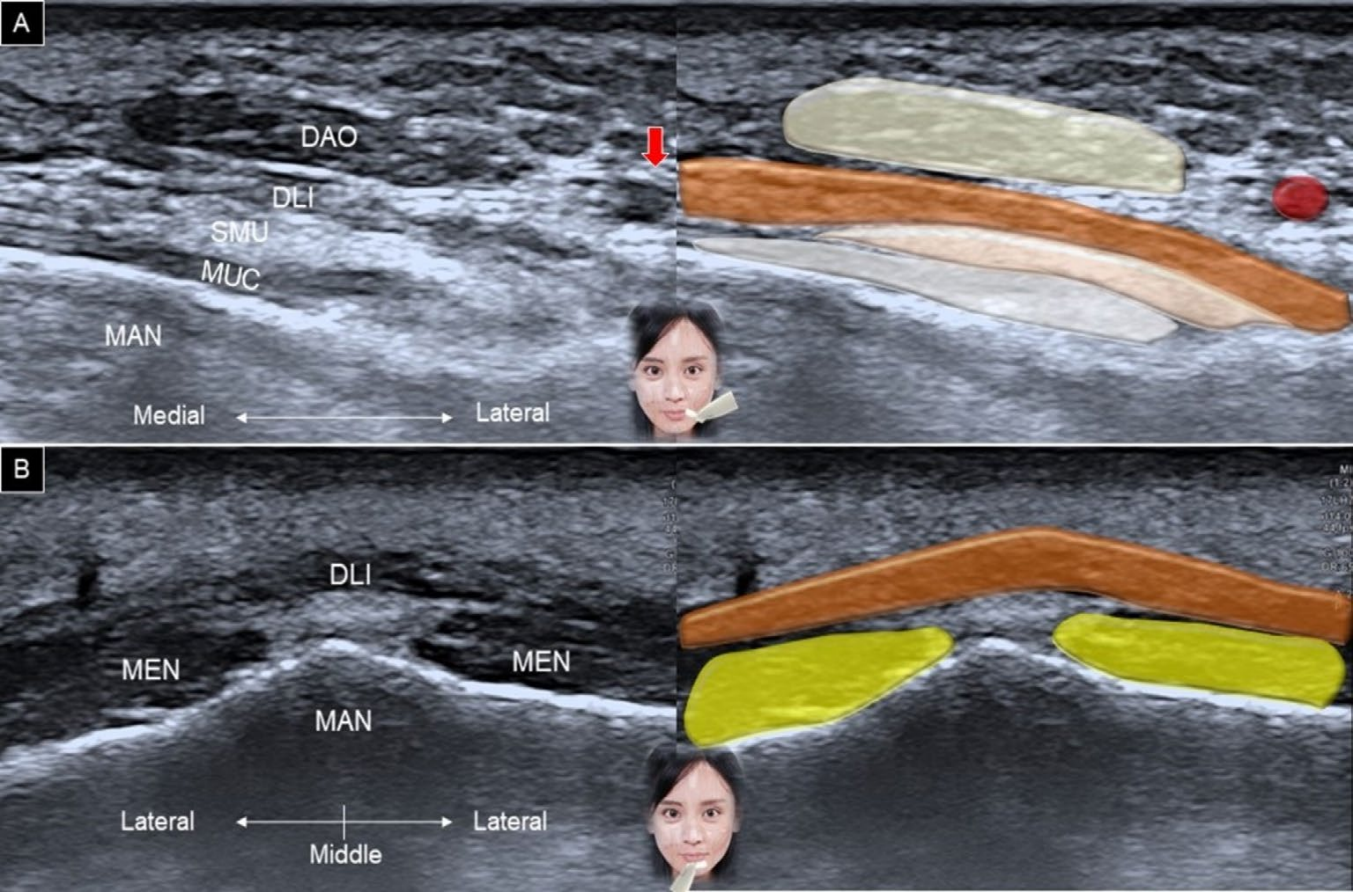
Ultrasound imaging and schematic illustration of the depressor anguli oris, depressor labii inferioris (A), and mentalis (B) muscles. DAO, depressor anguli oris; DLI, depressor labii inferioris; MAN, mandible; MEN, mentalis; MUC, oral mucosa layer; SMU, submucosal layer; man, mandible; red arrow, facial artery.

**FIGURE 13:**
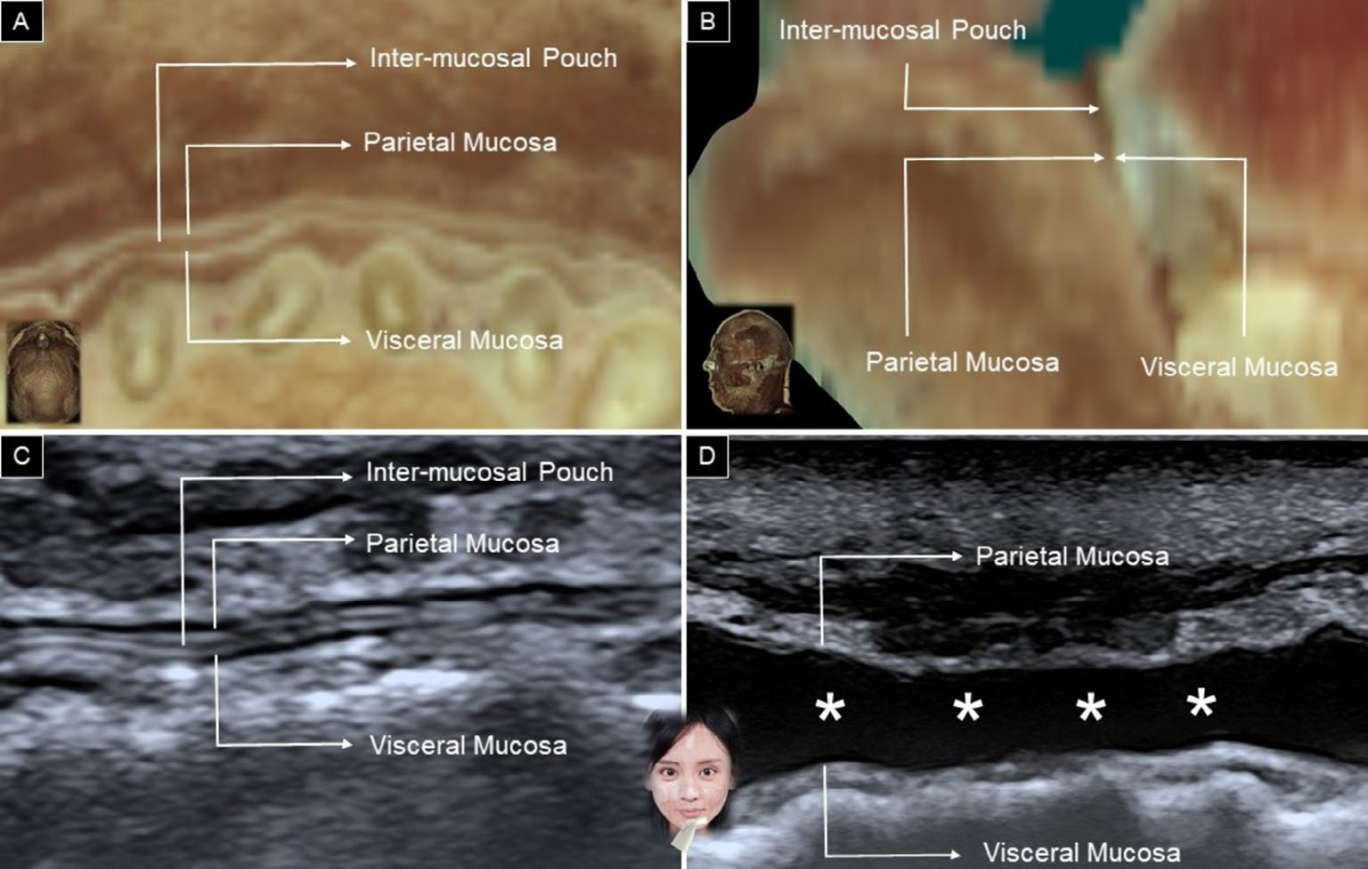
Cadaveric horizontal (A) and sagittal (B) dissections of the inter‐mucosal pouch, along with its ultrasound images in the horizontal plane before (C) and after (D) dilatation with water (asterisk). Cadaveric images are adapted from the Visible Human Project of the National Library of Medicine. Excerpts from these images, featured in the VH Dissector, are used with permission from Touch of Life Technologies Inc.

Rotating the other end of the transducer toward the mandibular angle allows identification of the depressor anguli oris muscle, with the depressor labii inferioris situated underneath (Figure 12A). Additionally, positioning the transducer horizontally along the labiomental crease enables visualization of the mentalis muscle in a short‐axis view, with the depressor labii inferioris lying above the mentalis muscle (Figure 12B). When scanning the lower lip region, the mucosal layer might be mistaken for the depressor labii inferioris. The sandwich‐like structure formed by the parietal mucosa, inter‐mucosal pouch, and visceral mucosa may resemble the fibrillar pattern of a muscle (Figure 13A,B). To differentiate between the inter‐mucosal pouch and the adjacent depressor labii inferioris muscle, the patient is asked to hold water in the mouth, expanding the former anatomical area (Figure 13C,D).

## 5 Filler Injections

While most complications from lip filler injections are minor and easily addressed, more serious issues such as vascular compromise, infections, and delayed‐onset nodules can severely impact both the aesthetic results and patient well‐being [22]. These significant complications often necessitate more invasive treatment approaches.

Utilizing US guidance can help in preventing these complications through vascular mapping and filler identification. It also aids in assessing problems such as intravascular embolus—an unintended injection of filler material into a blood vessel that can block blood flow, leading to tissue ischemia or necrosis, and requiring prompt intervention to restore circulation. Additionally, US can detect external vascular compression, which occurs when filler material compresses the nearby blood vessels, potentially impeding blood flow. By using real‐time imaging, US guidance can assist in managing these complications with precise identification of the location/extent of the filler, ensuring safer and more effective treatment by administration of hyaluronidase or other therapeutic injections.

**FIGURE 14:**
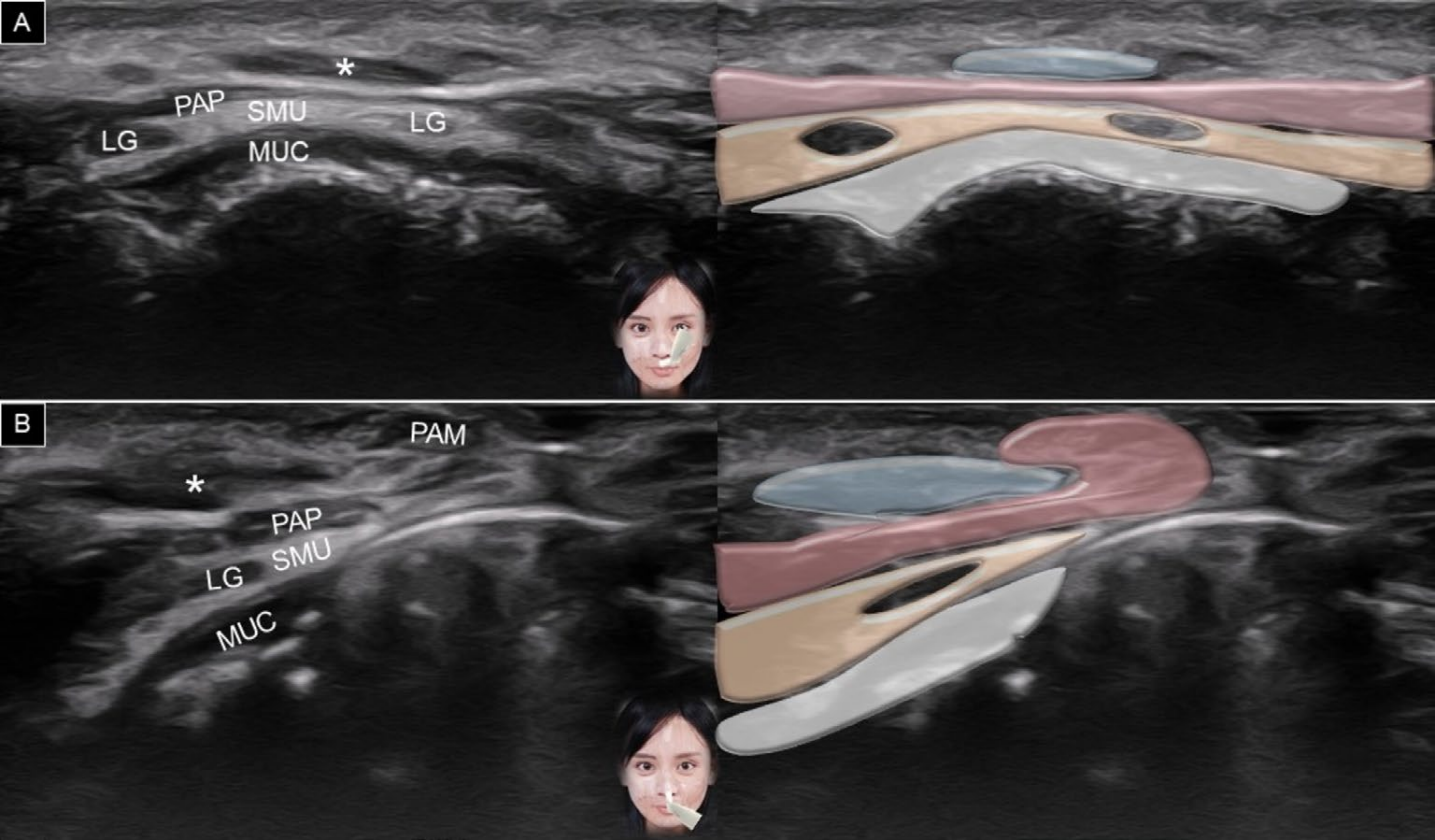
Ultrasound imaging and schematic illustration of the upper lip following hyaluronic acid filler injection, presented in both horizontal (A) and sagittal (B) planes. LG, labial gland; MUC, oral mucosa layer; PAM, pars marginalis; PAP, pars peripheralis; SMU, submucosal layer, asterisk, hyaluronic acid filler.

Before injecting fillers, physicians should perform a scout scan of the lip by placing the transducer in the sagittal plane along the lip's midline and sweeping it toward the bilateral oral commissures. The first goal is to identify any local haziness or hypoechoic region from previous hyaluronic acid injections (Figure 14), if any. The second goal is to map the vessels to detect any unusual paths. Since the superior and inferior labial arteries typically follow submucosal courses, Lee et al. [19] recommend a very superficial injection, just slightly below the dermis. In such cases, real‐time US‐guided injection may not be necessary, as the transducer could obstruct the needle's movement within the lip. However, if the injection (e.g., hyaluronidase) aims to dissolve intra‐lip nodules caused by previous improper filler injections, real‐time US guidance is essential to locate the accumulated areas of hyaluronic acid [23].

## 6 Conclusion

This review revisits the lip anatomy, offering an overview of local US applications and a systematic approach to lip sonography. US imaging and guidance effectively delineate the soft tissues, vasculature, and relevant muscles of the lip region—holding significant potential to prevent unwanted complications during aesthetic filler injections.

## Author Contributions

Conceptualization, W.‐T.W. and K.‐V.C.; methodology, K.‐V.C., H.‐C.C.; software, H.‐C.C. validation, K.M., V.R. and L.Ö.; writing – original draft preparation, K.‐V.C.; funding acquisition, K.‐V.C. All authors have read and agreed to the published version of the manuscript.

## Consent

Patient consent was obtained.

## Conflicts of Interest

The authors declare no conflicts of interest.

## Data Availability

Data are contained within the main text of the manuscript.
